# Real-Time Bladder Lesion Registration and Navigation: A Phantom Study

**DOI:** 10.1371/journal.pone.0054348

**Published:** 2013-01-24

**Authors:** Michelle Agenant, Herke-Jan Noordmans, Wim Koomen, J. L. H. Ruud Bosch

**Affiliations:** 1 Department of Urology, University Medical Centre Utrecht (UMCU), Utrecht, The Netherlands; 2 Department of Medical Technology and Clinical Physics, University Medical Centre Utrecht (UMCU), Utrecht, The Netherlands; 3 Engineering, Personal Space Technologies (PS-Tech), Amsterdam, The Netherlands; 4 Department of Urology, University Medical Centre Utrecht (UMCU), Utrecht, The Netherlands; The University of Hong Kong, Hong Kong

## Abstract

**Background:**

Bladder cancer is the fourth most common malignancy in men, with a recurrence rate of 33–64%. Tumor documentation during cystoscopy of the bladder is suboptimal and might play a role in these high recurrence rates.

**Objective:**

In this project, a bladder registration and navigation system was developed to improve bladder tumor documentation and consequently increase reproducibility of the cystoscopy.

**Materials/Methods:**

The bladder registration and navigation system consists of a stereo-tracker that tracks the location of a newly developed target, which is attached to the endoscope during cystoscopy. With this information the urology registration and navigation software is able to register the 3D position of a lesion of interest. Simultaneously, the endoscopic image is captured in order to combine it with this 3D position. To enable navigation, navigational cues are displayed on the monitor, which subsequently direct the cystoscopist to the previously registered lesion. To test the system, a rigid and a flexible bladder phantom was developed. The system's robustness was tested by measuring the accuracy of registering and navigating the lesions. Different calibration procedures were compared. It was also tested whether system accuracy is limited by using a previously saved calibration, to avoid surgical delay due to calibration. Urological application was tested by comparing a rotational camera (fixed to the rotating endoscope) to a non-rotational camera (dangling by gravity) used in standard urologic practice. Finally, the influence of volume differences on registering and navigating was tested.

**Results/Conclusion:**

The bladder registration and navigation system has an acceptable accuracy for bladder lesion registration and navigation. Limitations for patient determinants included changes in bladder volume and bladder deformation. *In vivo* studies are required to measure the effect of these limitations and functionality in urological practice as a tool to increase reproducibility of the cystoscopy.

## Introduction

In western countries, bladder cancer is the fourth most common malignancy in men [1], [2].

Most bladder tumors are confined to the mucosa (stage Ta or CIS) or submucosa (T1). These are defined as non-muscle-invasive bladder cancer (NMI-BC) and are diagnosed by inspection of the entire bladder wall during a cystoscopy, mostly using a 30° endoscope [3]. Treatment consists of transurethral resection of the bladder tumor (TURBT) in which most of the tumors are completely resected. Nevertheless, recurrence and residual tumors are common issues that can lead to disease progression, even with additional intravesical (chemo- or immuno-) therapy [4]–[7]. For T1 or high-grade NMI-BC, recurrence rates of 33–64%, residual tumor rates of 28–37% and progression rates of 11–27% have been reported [2], [8]–[10]. If there is a high risk for recurrence or residual tumor after initial resection, a re-TURBT is performed after 6 weeks to evaluate the completeness of resection or to define the total tumor invasion [3], [11].

There are two problems associated with the current diagnostic approach. First, there is no certainty that the whole bladder has been inspected during a cystoscopy as the urologist might overlook a small part of the bladder that contains a suspect lesion.

Second, conventional tumor documentation is suboptimal, which is a major limitation for patient follow-up and treatment. The tumor location is currently registered in the patient's medical record by making a provisional hand-made drawing of lesions on a bladder schedule. Subsequent follow-up cystoscopy, or cystoscopy during TURBT, is then performed with specific attention paid to these marked areas on the schedule. However, this approach lacks documentation accuracy because of the inter- and intra-urologist variability related to the drawing and interpretation. In addition, information on the size and shape of the tumor is inadequate. Although, endoscopic images containing that information can be saved, they still lack information on the location of the lesion.

To overcome these problems, we developed a bladder registration and navigation system. This system registers which parts of the bladder have been inspected during a cystoscopy. Furthermore, it allows to capture the 3D position of endoscopic images to generate a panoramic bladder wall overview. This enables a cystoscopic comparison of new and previously performed inspections. This serves to improve patient follow-up and increases the reproducibility of the cystoscopy.

The aim of this study is to demonstrate the first concept of the bladder registration and navigation system. It is a stage 0 preclinical study, according to the IDEAL recommendations [12]–[14]. Tests are performed to analyze the technical determinants of the system, i.e. the stability of registering a location, navigating back to that location, how and when to calibrate the system and the influence of the dangling endoscopic camera. Secondly, as navigation in the bladder places extra demands on the system, (e.g., the deformable nature of the bladder), additional tests are performed to study these effects.

## Materials and Methods

### Bladder navigation system

**Figure 1:**
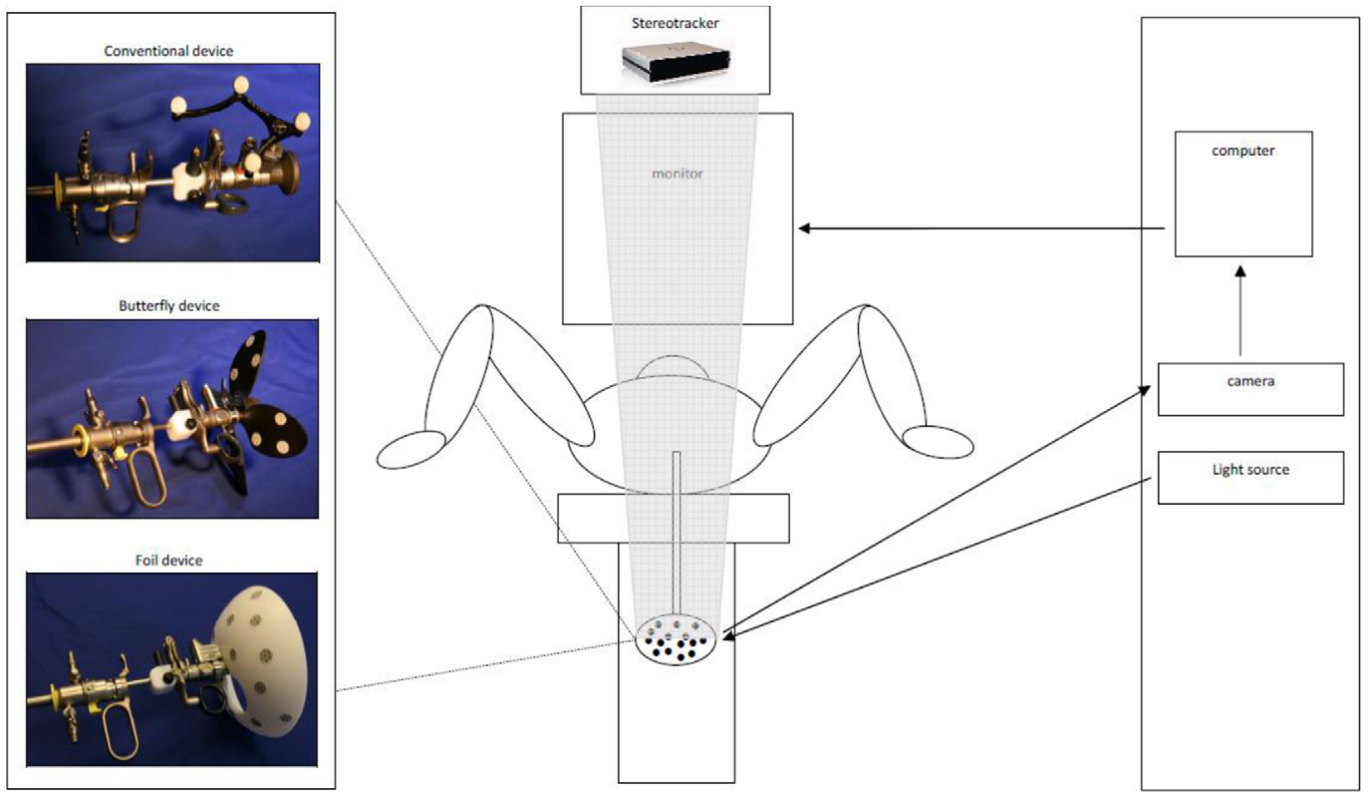
Schematic bladder navigation system. The typical position of a patient during cystoscopy or TURBT, with the legs in stirrups. The cystoscope set is connected to the light source, endoscopic camera and a tracking target (conventional, butterfly or foil) The camera sends image data to a computer that converts the analogous imaging data to digital imaging information. This is combined by a computer with positional information of the tracking target, which is read by the stereo-tracking camera. Standard endoscopic images edited with navigational directions are displayed on the monitor.

The bladder navigation system consists of a conventional cystoscopic system equipped with 3 extra components, to register and navigate positions in the 3D space inside the bladder (Figure 1). The first component is a tracking target connected to the endoscope which moves together with the scope. The second component is a stereotracker which can be placed above the standard endoscopic monitor and subsequently measures the 3D position of the tracking target. The third component is a computer that converts analog imaging data of the endoscopic camera to digital data and combines it with the 3D position information of the endoscope tip, which is generated from the stereotracker and tracking target.

#### Tracking target

First, we experimented with conventional tracking targets, where near-infrared reflecting spheres are mounted on a frame (Figure 1). Although conventional tracking targets are easily clamped to an endoscope, two problems arose which severely hampered their use for a urological application. First, while rotational movement is essential during cystoscopy and TURBT, the tracking camera lost sight of the conventional tracking target when the endoscope was turned upside down. Secondly, the target was easily lost by the system because the sight path from the camera to the tracking target was often disturbed by aspects required in the urological application i.e., the light cable, the head of the surgeon, and the legs of the patient that are placed in stirrups.

To overcome these two problems, we devised two new tracking targets i.e., the butterfly target and the foil target (Figure 1). Both of these are equipped with retro-reflective patches instead of the reflecting spheres. In addition to being constantly visible by the tracking camera, we stipulated that the target could be clamped to the endoscope in one unique way. In this way, a previously obtained calibration can be loaded into the software, avoiding surgical delay due to calibration.

In a dedicated test (results not shown), the foil target outperformed the conventional and butterfly design in overall visibility and user friendliness; therefore, this foil target was used for all subsequent tests.

#### The stereotracker

For this project we used a *PS-Tech* stereo-tracking camera, which is a motion-based tracker that determines the position and orientation (POSE) of arbitrary objects in a coordinate system relative to a known origin. In this stereotracker two major components can be distinguished:

*The infrared lighting panel* illuminates the workspace with near-infrared light.
*Two infrared cameras* observe the tracking space with a frequency of 55 Hz, each from a slightly different viewpoint. This infrared illumination is synchronized with the cameras enabling capture of the reflected near-infrared light from the retro-reflective patches of the target.

#### Computer software

For registration, 3D positions are captured by a medically-approved computer during cystoscopy in a shared coordinate system, which indicates the relative 3D distance to each other in space.

To enable navigation, the cystoscopist is guided to the registered 3D positions by multiple directional cues, i.e. arrows and point markers. The arrows indicate the 3D direction in which the cystoscopist should direct the scope, and the point markers are virtual representations of the registered points that increase in size when the tip of the scope approaches the registered 3D location. To achieve this, the endoscopic video stream is captured by the computer and the navigation cues are superimposed over the digitized endoscopic video stream. Subsequently, the combined video stream is transferred to a second endoscope monitor that shows the augmented reality view. This monitor is placed next to the original endoscopic monitor, so that both monitors are shown during cystoscopy. To enable this, the entire endoscopic imaging and tracking chain is modeled.

First, the 3D position of the endoscope and attached target is determined with the use of triangulation methods and by measuring the 3D Euclidean transformation of the target [15].

Second, to achieve the augmented reality view, it is necessary to model the projective properties of the endoscope using two parts.

The *intrinsic* part is based on the pinhole camera model and contains the parameters that are invariant under motion [16], [17]. It is represented by the 3 by 4 matrix, which is called **K**:
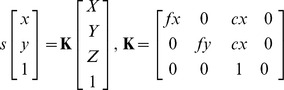

*X*, *Y* and *Z* are elements of a 3D point defined in camera space, *x* and *y* are the coordinates of the projected 3D point and *s* is an arbitrary scale factor. The elements *f_x_* and *f_y_* in matrix **K**, describe the focal length in pixels along the x- and y-axes and *c_x_* and *c_y_* indicate the location of the principal point.
The *extrinsic* part describes the transformation from world space coordinates to camera space coordinates. In our model it is defined by two matrices: **A** and **T**. Matrix **A** is a 4 by 4 Euclidean transformation matrix that is completely defined by the POSE of the tracking target which is attached to the endoscope in real-time. This results in a change of parameters, when the endoscope is moved through the workspace. Because the coordinate frame of the tracking target and the coordinate frame of the endoscope camera do not align, we have to incorporate an offset transformation into our model: a 4 by 4 Euclidean transformation matrix **T**. This matrix transforms the 3D coordinates from the coordinate space of the tracking target to the endoscope's camera coordinate space.

The full cystoscopic projection model is defined as:
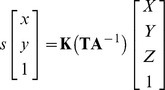
Matrix **T** and **K** are fixed and matrix **A** is updated every frame, using parameters that are retrieved from the stereo-tracking camera.

#### Calibration method

All parameters of the cystoscopic projection model are determined using one integrated calibration method. Several snapshots (at least two) are taken by pointing the endoscope to a planar checkerboard-patterned calibration board, which itself is tracked using retro-reflective patches. The POSE of the calibration board and the tracking target are saved, together with a set of 2D points extracted from the endoscopic camera image. From the entire set of 2D points the intrinsic parameters are calculated; simultaneously, the extrinsic camera parameters are determined for every snapshot, both by using Zhang's calibration method [17].

In addition to the linear pinhole model, the optics of the endoscope show significant amounts of non-linear distortion. For this reason we modeled the non-linear distortion using a 5-parameter version of the Brown distortion model (3 for radial distortion and 2 for the tangential distortion) [18].

Offset matrix T can easily be calculated as the relation between the pose of the calibration board, tracking target and the endoscopic camera, and is known from Zhang's calibration procedure.

#### Registering locations

To register locations of interest, the user moves the endoscope until the tip is touching the location of interest. By pushing a pedal or a key on the computer, the 3D position of the tip of the endoscope that allocates a lesion of interest is stored in the system. At the same time the endoscopic image is captured and stored as digital image (PNG) to enable generation of panoramic overviews and comparison with previously obtained cystoscopy information.

#### Navigating locations

The cystoscopist effectuates navigation by moving the tip of the endoscope to a previously registered 3D position, by navigation cues that denote the direction and distance to that position. To enable that, the 3D position of the location is transferred to the current image space.

### Phantoms

**Figure 2:**
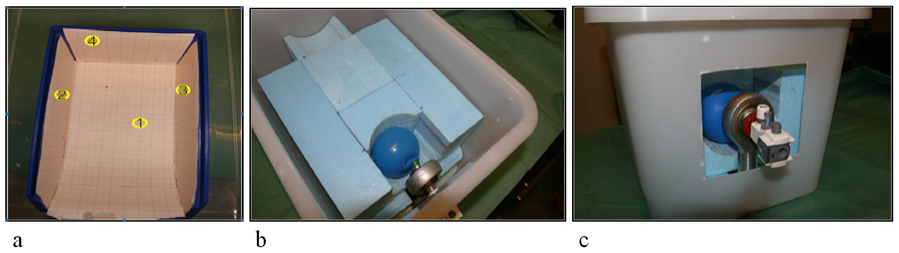
Phantoms. a. Is the box phantom with four lesions drawn on the inside; b. and c. are the balloon phantom with four lesions drawn inside the balloon, coupled to the trocard holder.

**Figure 3:**
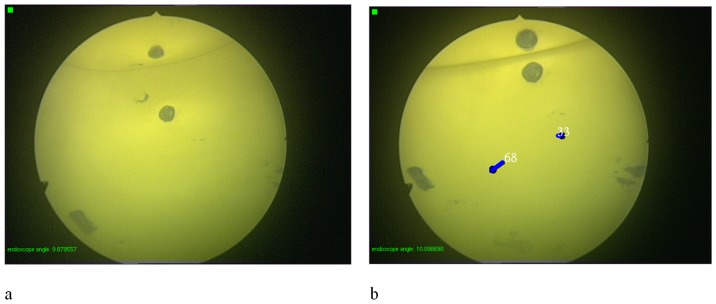
Endoscopic balloon phantom. Views through the endoscope before and after registering lesion number 68 and 33. Note that one lesion is mirrored in the air bubble.

Because no commercial functional phantom was available to measure the accuracy of registering and navigating lesions without fluid leakage during introduction of the scope, we designed two phantoms. The first is a dry ‘box phantom’ with graph paper on the inside (Figure 2A) and the second a ‘balloon phantom’ which can be filled with different volumes of water (Figure 2B and 2C). The endoscope is introduced through openings in the phantom that simulate the bladder neck, which connects the ‘bladder’ (box or balloon, respectively). The ‘balloon phantom’ contains a luer lock trocar holder connection in the opening, which provides a fluid-filled balloon without leakage. Similar to the original anatomy where bony structures and the rectum surround the bladder, the balloon expands specifically to the ventral side because sponge material surrounds the other parts of the balloon. Adjusting the volume of water in the balloon simulates bladder content increase/decrease, and deformation. In both ‘bladders’, four ‘lesions’ were drawn (on the floor, right wall, left wall and back wall), to facilitate 3D position registering and navigation. A lesion in the dome of the balloon was omitted, because the thin balloon wall deformed very easily at the ventral side when the scope touched the wall, due to the surrounding air instead of sponge material. Therefore, these measurements are incomparable to the stiffer real bladder. An example of registering the lesions and which hints are shown for navigation is shown in Figure 3A–B.

### Tests

#### Technical determinants

All technical determinant tests were performed in the box phantom to test the robustness of the system itself.

The *registering-accuracy* of the system was evaluated after conventional calibration with 24 snapshots. By registering each lesion 16 times, 64 3D positions in the bladder were recorded. The mean position per lesion was calculated. Then, Euclidean distance between each registered 3D point and the mean 3D point was calculated, per lesion.

*Navigation feasibility* was tested in the box phantom after conventional calibration with 24 snapshots. Each registered 3D position (one per lesion) was navigated 16 times by the cystoscopist, based on navigation cues on the monitor in the absence of endoscopic images. At the same time, the researcher observed another monitor that displayed both the navigation cues and the endoscopic imaging. When the cystoscopist finished navigation, the researcher subsequently documented whether the navigation was successful by designating whether the lesion was ‘on’ or ‘off’ screen.

To determine the minimum number of snapshots required for a functional calibration procedure, the registration-accuracy test was repeated 4 times; the first after standard calibration with 24 snapshots, which was then compared to calibrations using 18, 12 and 6 snapshots in the box phantom, respectively. We call this the *snapshot-calibration* test. The mean 3D position of each lesion of the standard calibration (24 snapshots) was calculated and the Euclidean distance between each registered 3D point and the mean standard 3D position was measured, per lesion.

To test whether a previously obtained calibration can be loaded into the software to avoid surgical delay due to calibration, the robustness of the target clamp is tested in the *calibration-requirement* test in the box model. After calibration using 24 snapshots, the back lesion was registered 16 times, then the cystoscope set and target were disassembled and reassembled 19 times and, each time, the registration was repeated 16 times. The mean 3D position of the set just after calibration, without taking the cystoscope set and target apart, was calculated and the Euclidean distance between each registered 3D point to that mean 3D point was calculated.

To assess whether an endoscope camera dangling to gravity was properly modeled by the software, *the rotation-correction* test was performed by repeating the navigation-feasibility test twice. First with the camera fixed to the endoscope (i.e. the conventional navigation system) and secondly with a dangling camera as in standard urological practice and, subsequently, a software correction, the rotation correction was investigated.

#### Patient determinants

Both of the patient determinant tests were performed in the balloon phantom, to enable analyses of the influence of volume differences in the bladder.

The influence of changing volume on the registering accuracy was verified (*registering-volume-influence* test). The registering-accuracy test was repeated 7 times using different volumes of water as follows: first the volume was stepwise decreased in increments of 60 cc (i.e. 480-420-360-300 cc) and then stepwise increased (i.e., 360-420-480 cc). A mean 3D position for the balloon filled with 300 cc water was calculated and the Euclidean distance between each registered 3D point and that mean 3D point was calculated, per lesion.

Finally, the influence of changing the volume was also tested on navigation, by repeating the navigation-feasibility test (*navigation-volume-influence* test). Lesions were registered in a balloon filled with 420 cc water and the registered 3D positions were navigated, as described in the navigation-feasibility test. Navigation was first performed within the same volume and then again after reducing the volume to 300 cc.

#### General conditions

All tests were performed in an operating room on a surgical table with clinical cystoscopic instruments *(Storz)*. The test phantoms were strapped to the table, to prevent movement of the phantom itself. All registration and navigation characteristics were recorded and the mean and spread of measure sets were calculated.

## Results

### Technical determinants

The mean Euclidean distances and standard deviations of all registration tests are depicted in Table 1 for each location and for all the locations together. Table 1 also includes the percentages of the correctly navigated lesions for the navigation-feasibility test, the rotational-correction test and the navigation-volume-influence test.

**Table 1:** Results overview.

	Variables	Floor		Right wall		Left wall		Back wall		Total	
		Mean	*Std*	Mean	*Std*	Mean	*Std*	Mean	*Std*	Mean	*Std*
Registering-accuracy test (box/mm)		**3.8**	*3.0*	**2.6**	*1.0*	**2.5**	*2.6*	**3.0**	*2.2*	**3.0**	*2.3*
Navigation-feasibility test (box/%)		**100%**		**100%**		**100%**		**75%**		**93.8%**	
Snapshot-calibration test (box/mm)	6 snapshots	**3.5**	*1.3*	**4.5**	*1.5*	**2.9**	*0.5*	**5.2**	*0.8*	**4.0**	*1.4*
	12 snapshots	**3.1**	*0.3*	**2.4**	*0.3*	**2.9**	*1.0*	**3.6**	*0.8*	**3.0**	*0.8*
	18 snapshots	**0.9**	*0.2*	**0.6**	*0.4*	**1.8**	*1.0*	**0.8**	*0.6*	**1.0**	*0.8*
	24 snapshots	**0.5**	*0.4*	**0.8**	*0.4*	**1.4**	*1.0*	**0.4**	*0.2*	**0.8**	*0.7*
Calibration-requirement test (box/mm)	0×taken apart							**0.6**	*0.3*		
	1×taken apart							**6.3**	*1.7*		
	2×taken apart							**3.1**	*0.2*		
	3×taken apart							**4.9**	*0.4*		
	4×taken apart							**8.9**	*0.2*		
	5×taken apart							**4.9**	*0.4*		
	6×taken apart							**3.5**	*0.1*		
	7×taken apart							**4.5**	*0.2*		
	8×taken apart							**4.6**	*0.2*		
	9×taken apart							**5.0**	*0.2*		
	10×taken apart							**3.9**	*0.2*		
	11×taken apart							**2.8**	*0.7*		
	12×taken apart							**3.5**	*0.2*		
	13×taken apart							**3.4**	*0.2*		
	14×taken apart							**5.5**	*0.2*		
	15×taken apart							**4.5**	*0.8*		
	16×taken apart							**4.8**	*0.4*		
	17×taken apart							**3.1**	*0.6*		
	18×taken apart							**3.2**	*0.2*		
	19×taken apart							**2.6**	*0.1*		
Rotational-correction test (box/%)	Fixed	**100%**		**100%**		**94%**		**100%**		**99%**	
	Rotation	**100%**		**100%**		**100%**		**100%**		**100%**	
Registering-volume-influence test (balloon/cm)	480 cc	**0.9**	*0.4*	**2.9**	*0.5*	**4.1**	*0.7*	**4.3**	*0.2*	**3.1**	*1.5*
	420 cc	**0.8**	*0.4*	**1.9**	*0.3*	**2.9**	*0.5*	**3.7**	*0.2*	**2.3**	*1.2*
	360 cc	**0.5**	*0.2*	**0.7**	*0.2*	**0.8**	*0.3*	**1.5**	*0.4*	**0.9**	*0.5*
	300 cc	**0.3**	*0.3*	**0.3**	*0.2*	**0.4**	*0.2*	**0.5**	*0.2*	**0.4**	*0.3*
	360 cc	**0.4**	*0.3*	**0.5**	*0.1*	**0.5**	*0.2*	**0.5**	*0.2*	**0.5**	*0.2*
	420 cc	**0.5**	*0.1*	**0.9**	*0.3*	**0.6**	*0.3*	**0.5**	*0.1*	**0.7**	*0.3*
	480 cc	**0.6**	*0.2*	**1.2**	*0.2*	**1.0**	*0.4*	**0.5**	*0.4*	**0.8**	*0.4*
Navigation-volume-influence test (balloon/%)	420 cc	**81%**		**56%**		**50%**		**100%**		**72%**	
	300 cc	**94%**		**100%**		**81%**		**100%**		**94%**	

All tests results are shown numerically summarized. The mean and standard deviation of the Euclidean distance to the reference positions are depicted (of every 16 registrations per lesion and per variable). The navigation tests (including the rotational-correction test) do not show a mean distance, but a percentage of the correctly navigated lesions, that were ‘on screen’ after navigation (also of every 16 navigations per lesion and variable).

**Figure 4:**
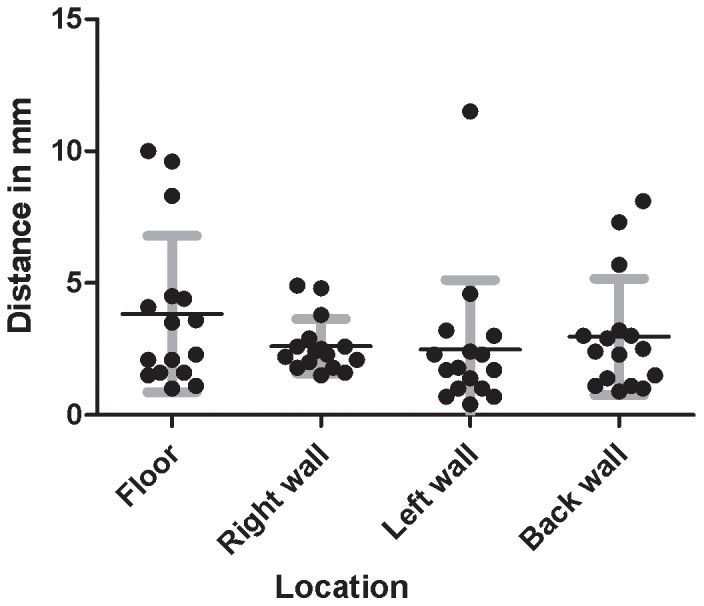
Registering-accuracy test. Mean and spread of the Euclidian distance of registered 3D points to the mean 3D point, per lesion.

The registering-accuracy test shows that the overall mean Euclidean distance to the mean position was 3.0 mm. The minimal and maximal mean distances were 2.4 mm for the left wall lesion to 3.8 mm for the floor lesion (Table 1 and Figure 4).

**Figure 5:**
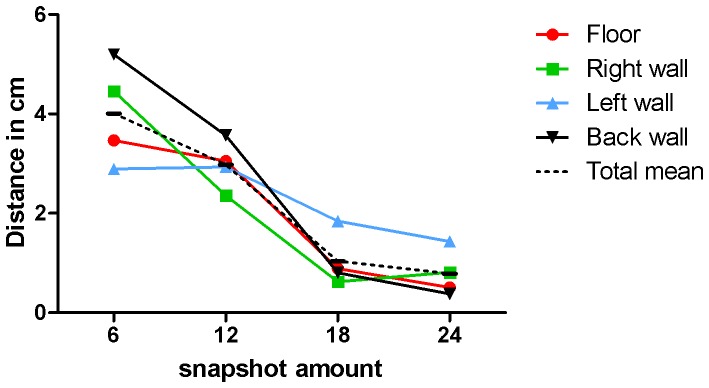
Snapshot-calibration test. Test to determine how many snapshots are minimally required to calibrate the urology navigation system. Errors clearly decrease using 18 or more snapshots.

The results of the navigation-feasibility test indicate that navigation within the box phantom is feasible (Table 1), with successful navigation in 93.8%. There was no clear difference between the lesions for navigation success. The results of the snapshot-calibration test, show that on average the registration error decreases when 18 or more calibration snapshots are used. The mean Euclidian distance for all lesions using 18 snapshots was 1.0 mm and standard deviation was 0.8 mm. (Table 1 and Figure 5).

**Figure 6:**
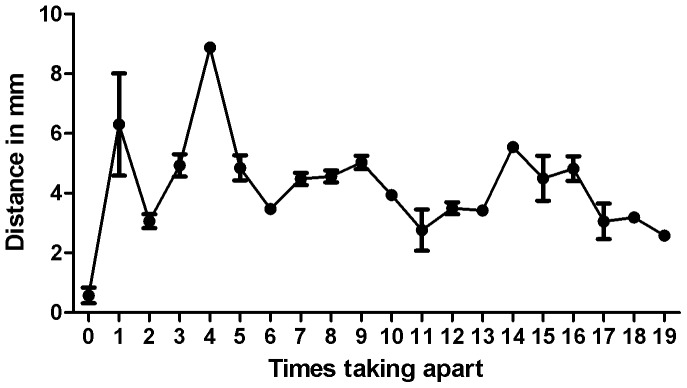
Calibration-requirement test. Test to assess whether a previously obtained calibration can be loaded into the software, to avoid delay in surgery due to calibration. The accuracy did not decrease after disassembling and reassembling the cystoscopy set and target several times.

The calibration-requirement test shows that there is no trend of differences in accuracy after disassembling and reassembling the instrument parts and target up to 19 times (Table 1 and Figure 6). Compared to the registrations of not taking apart the set with an Euclidian distance to the mean of the same set of 0,6 mm, the maximal Euclidian distance (8.9 mm) was measured after the set was taken apart the fourth time and the minimal Euclidian distance (2.3 mm) was measured after the set was taken apart the eleventh time,

The rotational-correction test reveals no clear difference in navigation accuracy when using the rotational software correction in the standard urologic system (dangling camera), compared to no rotational software correction in the conventional navigation system (fixed camera). Using the non-rotational setting, one lesion out of 64 navigations was off screen, whereas all lesions of the 64 navigations were on screen using the rotational setting.

### Patient determinants

**Figure 7:**
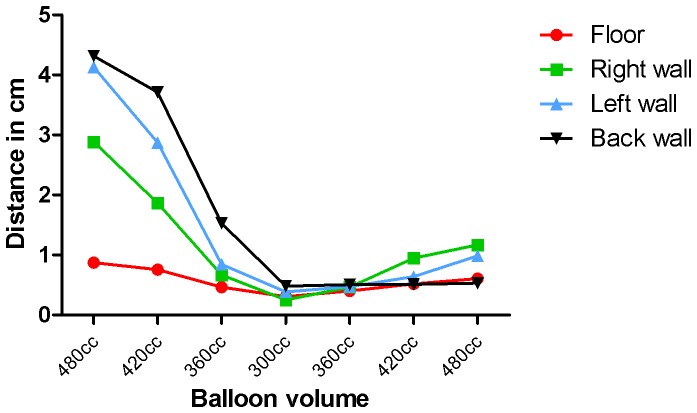
Registering-volume-influence test. Test to determine the influence of volume differences on the registering accuracy in the balloon phantom. For each measurement set per volume, the Euclidian distance between the registered 3D points and the mean registered 3D point of the 300 cc filled balloon were calculated per lesion.

The registering-volume-influence test shows a decrease in Euclidian distance when the balloon volume decreases (Table 1 and Figure 7). In contrast, the Euclidian distances were not increased after increasing the volume again.

The navigation-volume-influence test showed no significant difference between navigation success in the 300 cc compared to the 420 cc filled balloon (Table 1).

## Discussion

This study shows that our developed system is robust enough to register and navigate lesions. A 10 mm cut-off value for registering accuracy was determined based on the minimal field of view visible on screen during cystoscopy. The registration-accuracy test is representative of the system accuracy, which should be below or comparable to other errors in the system. The overall accuracy of 3 mm is therefore adequate. The outliers of this test (distances of 10 mm and 9.6 mm on the floor and 11.5 mm on the left wall) might be caused by interference of the light cable that might have changed its position with respect to the cystoscope set and target, as we have observed during the tests. The light cable consequently might block the retro-reflection of some patches to the stereotracker and if other patches reflect onto the stereo-tracking camera, the 3D position might deviate more than the characteristic spread of the system.

The navigation-feasibility test showed that navigation was successful, even in the worst-case scenario: navigation on navigational cues only, in the absence of endoscopic imaging. This is comparable to limited visibility in the bladder of patients with severe hematuria. Navigation of the back wall lesion was probably worse due to the smaller field of view (±1 cm) when the 30° endoscope is held against that lesion, compared to the side lesions that show a field of view of >2.5 cm.

To operate correctly, the system requires calibration that enables tracking by correctly assigning the visual tools on the augmented overlay. We showed that the overall tracking and image modeling can be calibrated in one step using several captures of a checker board. In practice, special care is required to capture snapshots from as many different angles as possible, to avoid extrapolation errors when a specific position has not been covered during calibration. Based on this assumption, 24 snapshots are sufficient for adequate calibration, which may be reduced to 18 snapshots to save time. We also investigated whether a previously obtained calibration could be loaded into the software to avoid the need for calibration during surgery. As the tracking target can be remounted with considerable precision, no additional errors were introduced after disassembling and reassembling. As a result, calibration may be performed outside the surgical flow. However, Figure 6 suggests that firmly connecting the cystoscope set and target is influenced by a learning curve of re-mounting and registering by the cystoscopist using the system. Furthermore, it should be mentioned that different scopes have different optical characteristics and consequently, that calibration should be performed for each endoscope separately. Finally, to make the registration reproducible, special care is required because the cystoscope set has some flexibility in assembly.

Furthermore, rotational corrections do not negatively affect navigation of registered lesions.

In contrast, the first patient determinants test shows that decreasing the balloon volume does affect the registering accuracy; this was expected because the 3D position of a lesion changes when the balloon volume decreases. Nevertheless, increasing the balloon volume showed no change in the registered 3D position of the lesion. This might be explained by the surrounding sponge material and the non uniform stretching of the balloon: i.e. when reducing the balloon volume the walls shrink and slide along the sponge material; conversely when increasing the volume again the balloon might stick to the sponge material and only extents to the ventral side where no sponge material is present. A limitation of this test is that the extension of the ventral side could not be proven because no lesion was drawn on that side.

Furthermore, volume reduction did not affect navigation accuracy. This was expected because during navigation, arrows indicate the direction to the registered 3D position, which is unchanged when using a smaller volume. Therefore, either way, the tip of the scope will reach the lesion. Influence of volume increase on navigation was not tested.

We present a newly developed system to register and navigate bladder lesions. Furthermore, we show that bladder tumors can be registered and subsequently navigated with acceptable accuracy in phantom models based on urologic conditions.

This system has additional diagnostic value and can be used together with other advanced optical techniques to improve tumor detection, e.g. photodynamic diagnosis (PDD), narrow band imaging (NBI) and optical coherence tomography (OCT) [19].

Because small lesions can be diagnosed by cystoscopy, this system allows to register the location and to navigate lesions of all sizes. This is in contrast to the virtual cystoscopy techniques (currently being developed) that detect and register only those tumors measuring ≥5 mm using CT [20]–[27], MRI [20], [28] and sonography [29], [30]. An advantage of these latter techniques is that invasion can be distinguished and therefore (in the future) a combination of both advantageous properties for bladder registration and navigation may be preferable.

In addition, unlike CT or MRI-based navigation techniques, no pre-operative imaging or anatomical or artificial landmarks (fiducials) are required [31].

Furthermore, the system allows direct feedback to the cystoscopist. The augmentation of endoscopic imaging results has only a few milliseconds time delay, which is very short compared to the mosaicking algorithms that are being developed to construct panoramic images of the bladder wall [32]–[37].

Other applications for navigation are cases with severe hematuria, where lesions can be easily found and quickly treated. During PDD, the endoscope is held closer to the bladder wall due to the blue light, which has low illumination power. Consequently, diagnosing multifocal tumors is improved because the system avoids the limitation of the small field of view.

The translation from the balloon phantom to the real bladder remains uncertain. Other studies have analyzed the influence of volume differences on the bladder structure and found bladder deformation [38], [39]. In our model, expansion of the bladder wall due to an increase in volume was not proportionate for all sides, probably due to pressure of the surrounding ‘tissues’. Therefore, predicting the 3D position changes of lesions due to volume changes in the bladder remains difficult.

Furthermore, the ease of use and duration of the surgical time, needs to be evaluated using this system.

We will perform an *in vivo* pilot study to test the feasibility of the bladder navigation and registration system, focusing on the 3D position change of different lesions (including the dome) after volume increase/decrease and bladder deformation. To analyze this using the current system, the bladder registration and navigation accuracy will be tested and compared using different fixed bladder volumes. This is a challenge during TURBT because the bladder is rinsed to prevent blood in the urine that limits vision. In the future, some analytical software adjustments might be developed when creating the bladder map for different volumes.

Registering the inspected parts of the bladder during cystoscopy will reduce the concern related to possibly overlooking a small part of the bladder that might contain a suspect lesion. Therefore, this system can be used as a quality control during urologic procedures; once feasibility has been demonstrated this should be tested. A prospective multi-center study is needed to measure the clinical feasibility of the bladder registration and navigation in urological practice and its inter-user variances. This proposed study will investigate whether the bladder registration and navigation system improves the reproducibility of the cystoscopy. Consequently, a reduction of residual and recurrent tumors due to better registration, follow up and more complete surgery of bladder lesions might be expected as a result. An improvement of disease-free survival will be the ultimate goal of implementing this clinical tool.

## Conclusions

The bladder registration and navigation system was developed to improve the reproducibility of the cystoscopy by improved bladder tumor documentation according to tumor size, number, shape and especially location, in real-time during a cystoscopy. It is hoped that this will lead to a reduction in residual and recurrent tumors and, consequently, to an improved disease-free survival.

Because the system successfully functioned in its form for urological purposes in phantom models, it can now be used in clinical trials.

*In vivo* tests will be performed at our department to examine feasibility for the urological clinic, focusing on the effects of volume differences and bladder deformation. Future studies are required to evaluate the ultimate goal: reduction in the number of residual and recurrent tumors and consequently improved disease-free survival.
