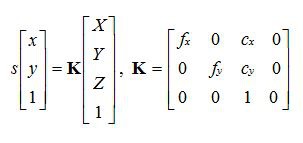# Correction: Real-Time Bladder Lesion Registration and Navigation: A Phantom Study

**DOI:** 10.1371/annotation/f86c89e9-af4e-4651-ad79-3f9d5b65db25

**Published:** 2013-10-24

**Authors:** Michelle Agenant, Herke-Jan Noordmans, Wim Koomen, J. L. H. Ruud Bosch

In the Materials and Methods section, there was an error in the second line, third to the right of the K matrix. The C_x_ should be C_y_. Please see the complete corrected equation here: